# Recent Insights into the Pathogenesis of Type AA Amyloidosis

**DOI:** 10.1100/tsw.2011.64

**Published:** 2011-03-07

**Authors:** J. C. H. van der Hilst

**Affiliations:** ^1^ Department of Internal Medicine and Infectious Diseases, University Medical Centre Utrecht, Utrecht, The Netherlands; ^2^ Department of Infectious Diseases, Jessa Hospital, Hasselt, Belgium

**Keywords:** amyloidosis, heat shock proteins, isoprenoid metabolism, serum amyloid A, autoinflammatory diseases

## Abstract

The amyloidoses are a group of life-threatening diseases in which fibrils made of misfolded proteins are deposited in organs and tissues. The fibrils are stable, insoluble aggregates of precursor proteins that have adopted an antiparallel β-sheet structure. In type AA, or reactive, amyloidosis, the precursor protein of the fibrils is serum amyloid A (SAA). SAA is a 104-amino-acid protein that is produced in the liver in response to proinflammatory cytokines. Although the protein that is produced by the liver contains 104 amino acids, only the N-terminal 66–76 amino acids are found in amyloid fibrils. Furthermore, SAA has been shown to have an α-helical structure primarily. Thus, for SAA to be incorporated into an amyloid fibril, two processes have to occur: C-terminal cleavage and conversion into a β-sheet. Only a minority of patients with elevated SAA levels develop amyloidosis. Factors that contribute to the risk of amyloidosis include the duration and degree of SAA elevation, polymorphisms in SAA, and the type of autoinflammatory syndrome. In the Hyper-IgD syndrome, amyloidosis is less prevalent than in the other autoinflammatory diseases. *In vitro* work has shown that the isoprenoid pathway influences amyloidogenesis by farnesylated proteins. Although many proteins contain domains that have a potential for self-aggregation, amyloidosis is only a very rare event. Heat shock proteins (HSPs) are chaperones that assist other proteins to attain, maintain, and regain a functional conformation. In this review, recent insights into the pathogenesis of amyloidosis are discussed, in addition to a new hypothesis for a role of HSPs in the pathogenesis of type AA.

## INTRODUCTION

The amyloidoses are a group of life-threatening diseases in which fibrils made of misfolded proteins are deposited in organs and tissues. The fibrils are stable, insoluble aggregates of precursor proteins that have adopted an antiparallel β-sheet structure. The process of fibril formation is a nucleation-dependent process: a critical concentration of β-sheet–formed molecules is necessary below which no aggregation will occur. Following a lag phase during which a nucleus is formed, there is rapid expansion of fibrils that are deposited in the extracellular matrix. In the extracellular space, the fibrils become resistant to degradation by the binding of proteoglycans and proteins, such as heparan sulfate and serum amyloid P (SAP)[1]. To date, at least 23 precursor proteins have been described that are capable of adopting a β-sheet configuration and form a fibril[2]. These include the Aβ peptide that is the major constituent of senile plaques of Alzheimer's disease, the short chain of gammaglobulin in amyloid light chain (AL) amyloidosis, and the β2 microglobulin that is found in amyloid deposits in patients receiving hemodialysis.

## CLINICAL FEATURES OF TYPE AA AMYLOIDOSIS

Clinical symptoms of reactive (type AA) amyloidosis are dependent on the organ involved. Virtually any organ can be affected by amyloid deposits, but the kidneys are the most frequently involved. Type AA amyloidosis, therefore, usually presents as proteinuria with or without renal impairment. Renal involvement is found in >90% of patients[3]. If the underlying inflammatory process cannot be controlled, renal failure will ensue. Gastrointestinal involvement, presenting as diarrhea and malabsorption, is seen in about 20% of patients. This has become more common with the availability of hemodialysis and renal transplantation, which has increased the life expectancy and thus duration of amyloid accumulation. Amyloidotic goiter, hepatomegaly, splenomegaly, and polyneuropathy are less frequently encountered features of type AA amyloidosis[4,5].

A diagnosis of amyloidosis can be confirmed by detection of amyloid fibrils in biopsy material. Amyloid fibrils stain with Congo Red, giving a typical apple green birefringence under polarized light. Immunohistochemistry can subsequently differentiate between different types of amyloid[6]. AA amyloidosis is a severe condition with high mortality. The median survival shown in older studies was 24 to 53 months from time of diagnosis[3,7,8,9]. The progression of type AA amyloidosis and survival is strongly dependent on the ability to control the underlying inflammatory process. If the inflammation can be controlled, regression of amyloid mass can occur as well as a reduction or reversal of nephropathy[10].

## PATHOGENESIS OF TYPE AA AMYLOIDOSIS

In type AA, or reactive, amyloidosis, the precursor protein of the fibrils is serum amyloid A (SAA). SAA is a 104-amino-acid protein that is produced in the liver in response to proinflammatory cytokines. It can be up-regulated 1000-fold during inflammation[11]. SAA is found in the serum as an apolipoprotein. SAA has been implicated as an opsonin for Gram-negative bacilli[12], as a factor of leukocyte chemoattraction[13], and in cholesterol metabolism[14,15]. However, despite intense research efforts, the exact function of SAA remains unknown. The protein that is produced by the liver contains 104 amino acids. However, amyloid fibrils mainly consist of the N-terminal 66–76 amino acid fragments. Full-length SAA can be found, but is only a minor constituent of amyloid fibrils[16,17,18]. Furthermore, SAA has been predicted to have an α-helical structure primarily[19,20]. Thus, for SAA to be incorporated into an amyloid fibril, two processes have to occur. First, the C-terminal part has to be cleaved and then the molecule has to adopt a β-sheet configuration. Previous research has shown that this process of C-terminal cleavage and aggregation, which is often referred to as amyloidogenesis, takes place in macrophages[21,22]. SAA is rapidly taken up by macrophages and transported to the lysosomal compartment. Under normal conditions, SAA is completely degraded without the appearance of amyloid fibrils. However, in patients with amyloidosis, intermediate SAA products somehow appear that aggregate into fibrils. After deposition of these accumulated intermediates in the extracellular space, several glycosaminoglycans, SAP, and lipid components bind to the fibril, and confer resistance to proteolysis[23,24,25].

So far, it remains largely unknown why some patients with chronic inflammation develop amyloidosis, while other patients with seemingly the same prerequisites are spared from this complication.

Misfolding of proteins leading to aggregation poses a serious threat to normal cellular function. Recent research by Goldschmidt et al. showed that the majority of proteins contain segments that potentially can adopt a β-sheet configuration and self-aggregate into a fibril[26]. However, misfolding diseases, including amyloidosis, are only very rare events. This raises the question about what protects us from such misfolding diseases as amyloidosis.

This review gives an overview of both the mechanisms that increase the risk for developing amyloidosis and the mechanisms that protect us from developing this severe complication of chronic inflammation.

## FACTORS INFLUENCING THE RISK OF TYPE AA AMYLOIDOSIS

### Polymorphisms in Gene Coding for SAA

Polymorphisms in the gene coding for SAA1 influence the risk of amyloidosis. Two single nucleotide polymorphisms at exon 3 constitute three different isotypes: SAA 1.1 (Val52–Ala57), SAA 1.3 (Ala52–Ala57), and SAA 1.5 (Ala52–Val57).

Patients with a 1.1/1.1 genotype have a three- to sevenfold increased risk for amyloidosis[27,28,29,30].

### Matrix Metalloproteinases

Matrix metalloproteinases (MMPs) may play an important role in the pathogenesis of amyloidosis. MMPs are enzymes that modulate the extracellular matrix and are present in AA amyloid deposits[31]. SAA1 induces production of MMPs by mononuclear phagocytes and synovial fibroblasts[32,33]. Furthermore, MMP-1, -2, and -3 have been shown to degrade both SAA and AA fibrils *in vitro*[34]. Interestingly, these MMPs degrade SAA preferentially in the region spanning amino acids 52–58. We showed that the degradation of SAA by MMP-1 is dependent on the SAA1 isotype[35]. SAA1.5 is largely resistant to degradation by MMP-1, contrary to SAA1.1. This difference is determined by the capacity of MMP-1 to cleave at residues 57 and 58 of the protein, which are either Val57–Ile58 (SAA1.5) or Ala57–Ile58 (SAA1.1). The difference in capacity of MMP-1 to degrade the two isoforms of SAA1 could explain the differential risk of developing amyloidosis.

### Type of Fever Syndrome

Since SAA is an acute-phase protein, type AA amyloidosis is a complication of all chronic inflammatory conditions. Infectious disorders such as chronic osteomyelitis, tuberculosis, and malaria were previously the main causes of amyloidosis. However, in the Western world today, noninfectious inflammatory diseases are the leading cause. One group of conditions that are of particular increased risk for developing amyloidosis is the autoinflammatory diseases.

Autoinflammatory syndromes comprise a group of genetic diseases clinically characterized by recurrent febrile attacks lasting in length from a few days to a few weeks[36]. These episodes of fever are separated by symptom-free intervals of variable duration, although most patients have signs of continuous inflammation in laboratory evaluation. During attacks, patients have vigorous inflammation, with leukocytosis and elevated concentrations of acute-phase proteins, such as C-reactive protein (CRP) and SAA[36]. The best known of the autoinflammatory syndromes is familial Mediterranean fever (FMF), but three other entities have been identified as well: hyperimmunoglobulinemia D and periodic fever syndrome (HIDS), tumor necrosis factor (TNF) receptor-associated periodic syndrome (TRAPS), and the cryopyrin-associated periodic syndrome (CAPS), which encompasses Muckle–Wells syndrome (MWS), familial cold autoinflammatory syndrome (FCAS), and chronic infantile neurological cutaneous and articular syndrome (CINCA). Although all patients with chronic inflammatory conditions are at risk for developing type AA amyloidosis, the incidence varies widely between the different autoinflammatory syndromes. Before effective treatment was available, up to 60% of FMF patients developed amyloidosis. The reported incidence is about 25% in TRAPS patients and up to 35% of patients with MWS. However, in HIDS, <3% of patients develop amyloidosis[37]. Since HIDS is caused by a defect in mevalonate kinase, a potential role for the isoprenoid metabolism in the pathogenesis of AA amyloidosis was suggested[38].

## The Isoprenoid Metabolism

Mevalonate kinase is a major enzyme in the isoprenoid biosynthesis. In this metabolic pathway, cholesterol and a number of nonsterol isoprenoids are produced. These nonsterol isoprenoids include geranylgeranyl-phosphate and farnesyl-phosphate, which can bind covalently to an array of proteins, including Ras and Rho. Farnesyl binds to proteins that have a specific amino acid sequence at the C-terminus called a C*AAX* box. The isoprenylation is important in guiding the proteins to the proper membrane localization. Patients with HIDS seem to have all the prerequisites for developing amyloidosis[39]. They have a strong acute-phase response with high SAA concentrations during attacks, but also signs of subclinical inflammation when asymptomatic. However, the incidence of amyloidosis is remarkably low suggesting that the underlying genetic effect might offer protection to developing amyloidosis. HMG-CoA is the enzyme preceding mevalonate kinase in the isoprenoid pathway (Fig. 1). It can be inhibited by the cholesterol-lowering drug lovastatin. When human mononuclear cells are isolated and cultured *in vitro* in the presence of SAA and amyloid enhancing factor (AEF), amyloid fibrils appear after a few days[40]. Using this cell culture system of amyloidosis, we showed that the isoprenoid pathway influences amyloidogenesis[41]. Lovastatin reduces amyloid formation in a dose-dependent manner. This effect can be reversed by the addition of farnesol, implicating an important role of a farnesylated protein in the protection against amyloidosis. Furthermore, inhibiting the enzyme that attaches farnesyl-phosphate to target proteins also impairs amyloid production.

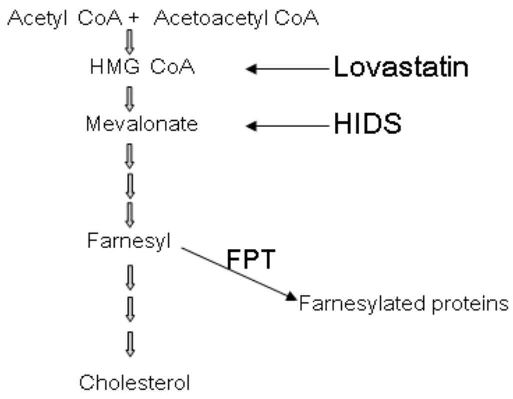

**FIGURE 1.** Schematic overview of the isoprenoid pathway. In the isoprenoid pathway, cholesterol is made from acetyl CoA and acetoacetyl CoA in several metabolic steps. Next to cholesterol, farnesyl is produced that can be covalently attached to proteins by the enzyme farnesyl protein transferase (FPT). In HIDS, mevalonate kinase is deficient, leading to a decrease of farnesylated protein. A deficiency of farnesyl can also be induced by inhibiting the enzyme HMG-CoA reductase, for example, by lovastatin.

A link between amyloid fibril production and isoprenoid metabolism is also seen in another form of amyloidosis, i.e., Alzheimer's disease, in which the cerebral plaques are composed of amyloid fibrils formed from the Aβ protein. This link is not only suggested by epidemiological studies[42,43,44,45], but also in an *in vitro* study with similarities to ours, i.e., Gellermann and colleagues[46], who found that inhibiting the isoprenoid pathway with lovastatin significantly reduced the formation of amyloid-like Aβ plaques by human macrophages.

Recently, a murine model of mevalonate kinase deficiency has been developed[47]. This model creates the opportunity to further investigate the impact of the isoprenoid pathway on amyloidogenesis.

## Amyloid Enhancing Factor

Another factor identified that influences amyloidogenesis both *in vitro* and in the animal models of AA amyloidosis is AEF. When mice are exposed to a continuous inflammatory stimulus, amyloid fibrils are formed in the spleen in response after a lag phase of 3–4 weeks. It has been known for a long time that this lag period can be shortened dramatically to 3 days by the simultaneous injection of extracts of spleen from amyloidotic mice[48]. Not only AEF generated from AA fibrils can thus shorten the lag period, but other types of amyloid fibrils have also been shown to act as AEF in AA amyloidosis, including AL amyloid fibrils[49]. Furthermore, the activity of AEF seems to be transmissible between animals. When mononuclear cells isolated from mice with detectable amyloid load are injected into healthy mice, the development of amyloidosis after an inflammatory stimulus is dramatically shortened[50]. Also, we showed that peripheral mononuclear cells derived from a patient with AL amyloidosis do not need AEF to induce amyloidosis in contrast to mononuclear cells from healthy volunteers[51]. The activity of this AEF has been shown to depend on small molecules with a β-sheet structure. AEF has been suggested to be a nidus for the generation and growth of amyloid fibrils. In a solution of an amyloid fibril protein above a critical concentration, a nucleus forms. AEF acts as a template for amyloid fibrillogenesis to begin, similar to a snowflake that starts growing from a speck of dirt[52].

## CATHEPSIN D PROTECTS AGAINST AMYLOID FORMATION

Chronic elevation of SAA is common in many patients with inflammatory diseases such as rheumatoid arthritis and autoinflammatory diseases. However, only a minority of patients develop amyloidosis. Normal degradation of SAA, without the formation of intermediate fragments that can adopt a β-sheet configuration, is essential in the prevention of amyloidosis. After endocytosis by mononuclear cells, the vesicles with SAA fuse with lysosomes. Here the degradation of SAA takes place. Many lysosomal enzymes, namely cathepsins, have been implicated in the degradation of SAA. Cathepsin B has been shown to cleave SAA *in vitro* with generation of an N-terminal fragment terminating at residue 76, analogous to the most common AA peptide, suggesting a role in amyloid pathogenesis[18,53]. However, inhibition of cathepsin B in a human cell culture system did not influence the degree of amyloid formation[54]. In addition, in cathepsin B, knock-out mice show a similar amount of amyloid deposition when compared to wild-type mice[55]. There is strong evidence that the amyloidogenic potential of SAA lies within the first 10–15 amino acids of the molecule[56,57]. Cathepsin D is able to degrade SAA in this critical region and there is accumulating evidence that cathepsin D is important in the normal, complete degradation of SAA. Cleavage of SAA by cathepsin D in the N-terminal region, therefore, would prevent the formation of N-terminally intact SAA peptides found in amyloid fibrils. Under conditions of reduced cathepsin D activity, other enzymes may have opportunity to degrade SAA at the C-terminus. In a mouse model of type AA amyloidosis, inhibiting cathepsin D by pepstatin resulted in significantly more deposition of amyloid. In addition, using a cell culture model of type AA amyloidosis, we showed that inhibition of cathepsin D in human monocyte results in a 43% increased accumulation of amyloid. Preliminary results show that rheumatoid arthritis patients that have developed amyloidosis have a decreased expression of cathepsin D compared to rheumatoid arthritis patients without amyloidosis (unpublished observation)

## THE ROLE OF HEAT SHOCK PROTEINS IN THE PROTECTION AGAINST AMYLOIDOSIS

Misfolding of proteins leading to aggregation poses a serious threat to normal cellular function. Recent research by Goldschmidt et al. shows that the majority of proteins contain segments that potentially can adopt a β-sheet configuration and self-aggregate into a fibril[26]. Selection processes during evolution have resulted in proteins without amyloidogenic capacity in their folded state. Amyloidogenic segments are folded within the molecule in a way that prevents self-aggregation. However, during protein synthesis, translocation processes, or as a result of environmental stresses, proteins are unfolded, increasing the risk that amyloidogenic structures are exposed. Chaperones are proteins that assist other proteins to attain, maintain, and regain a functional conformation. A subset of chaperones, the heat shock proteins (HSPs), were originally identified as proteins expressed in response to stress conditions, but it was later found that some HSPs are also constitutively expressed. The family of HSPs is classified into five major chaperone families based on their relative molecular weights: HSP110, HSP90, HSP70, HSP60, and small HSPs[58,59].

Experiments *in vitro* have shown that HSPs can prevent aggregation of other proteins as well as enhancing their refolding after heat or chemical denaturation[60,61,62]. A wide range of proteins is protected from aggregation during heat shock by HSPs. Since there is only a limited number of HSPs, this indicates that HSPs are rather promiscuous. The substrate properties, except for denaturation, that allow binding of HSPs are largely unknown.

Most HSPs are found in the cytosolic compartment of the cells, but HSPs can also be found in other compartments, including lysosomes.

### The Role of HSP70 in Folding of Proteins

Hsc70, a representative of the HSP70 family, is found both in the lysosomal membrane and in the lumen[63]. The membrane-bound form has a function in the translocation of proteins from the cytosol to the lysosomal lumen (chaperone-mediated autophagy). Cytosolic and lysosomal HSP70 can bind to partly unfolded proteins, preventing their misfolding and aggregation. This has broad substrate selectivity. The chaperone activity of HSP70 proteins is performed through an ATP-dependent cycle of peptide binding and release facilitated by cochaperone proteins such as GrpE and DnaJ (HSP40), among others[64].

### The Role of HSP40 in Folding of Proteins

HSP70 activity is tightly coordinated by the HSP40 cochaperone. HSP40 can bind to misfolded proteins and direct them to HSP70 for refolding or degradation. Furthermore, HSP40 stimulates HSP70 ATPase activity and stabilizes the HSP70-polypetide complex[65,66]. Furthermore, HSP40 can stabilize misfolded proteins independent from HSP70 activity.

Interestingly, HSP40 are unique in that they contain a C-terminal C*AAX* box that is post-translationally modified by farnesyl[67,68]. Farnesylation helps to localize a pool of HSP40 to the cytoplasmic face of the endoplasmic reticulum and is required for cells to survive heat stress[68]. Several recent studies show a fundamental role for HSP40 in amyloid-like protein synthesis in yeast[69,70,71]. The protective effects of HSP40 are dependent on farnesylation[71].

## CAN CHAPERONES PREVENT AMYLOIDOSIS?

Since misfolding followed by aggregation is the hallmark in all of the amyloidoses, a role for HSP can be hypothesized. Indeed, evidence is accumulating that HSPs play a role in the pathogenesis of different forms of amyloidosis. In Alzheimer’s disease, HSP B8, HSP27, HSP70, and HSP90 have all been shown to interact with the Aβ protein, preventing or reversing aggregation[15,72,73,74,75,76,77]. Furthermore, several HSPs can prevent *in vitro* amyloid formation from other precursor proteins[78,79,80,81]. Therefore, a decreased function or an improper localization of HSPs may lead to an increased risk of amyloidosis. Since the function of HSP40 has been shown to be dependent on farnesylation, the influence of the isoprenoid pathway on amyloidogenesis might be generated through interaction with HSP40. A deficiency of farnesyl leading to accumulation of HSP40 in the lysosomal compartment as seen in HIDS may be the explanation for the low risk of amyloidosis. Interestingly, a recent study by Tukaj et al. found that a significant number of patients with rheumatoid arthritis express anti-HSP40 antibodies[82]. Whether or not this contributes to the risk of developing amyloidosis remains unknown.

## CONCLUSION

Type AA amyloidosis is a life-threatening complication of inflammatory diseases. Polymorphisms in the gene coding for SAA and the isoprenoid pathway modulate the risk of developing amyloidosis. Evidence is accumulating that cathepsin D and HSPs protect against amyloidosis. The protective action of HSPs might be influenced by the isoprenoid pathway.
